# Effect of analgesic therapy on clinical outcome measures in a randomized controlled trial using client-owned dogs with hip osteoarthritis

**DOI:** 10.1186/1746-6148-8-185

**Published:** 2012-10-04

**Authors:** Sarah Malek, Susannah J Sample, Zeev Schwartz, Brett Nemke, Peer B Jacobson, Elizabeth M Cozzi, Susan L Schaefer, Jason A Bleedorn, Gerianne Holzman, Peter Muir

**Affiliations:** 1 Comparative Orthopaedic Research Laboratory, School of Veterinary Medicine, University of Wisconsin-Madison, Madison, WI, USA; 2 Abbott Laboratories, 100 Abbott Park Road, Abbott Park, IL, USA

**Keywords:** Dog, Hip, Osteoarthritis, Outcome measures, Clinical trial, Carprofen, Tramadol, ABT-116

## Abstract

**Background:**

Pain and impaired mobility because of osteoarthritis (OA) is common in dogs and humans. Efficacy studies of analgesic drug treatment of dogs with naturally occurring OA may be challenging, as a caregiver placebo effect is typically evident. However, little is known about effect sizes of common outcome-measures in canine clinical trials evaluating treatment of OA pain. Forty-nine client-owned dogs with hip OA were enrolled in a randomized, double-blinded placebo-controlled prospective trial. After a 1 week baseline period, dogs were randomly assigned to a treatment (ABT-116 – transient receptor potential vanilloid 1 (TRPV1) antagonist, Carprofen – non-steroidal anti-inflammatory drug (NSAID), Tramadol - synthetic opiate, or Placebo) for 2 weeks. Outcome-measures included physical examination parameters, owner questionnaire, activity monitoring, gait analysis, and use of rescue medication.

**Results:**

Acute hyperthermia developed after ABT-116 treatment (*P* < 0.001). Treatment with carprofen (*P* ≤ 0.01) and tramadol (*P* ≤ 0.001) led to improved mobility assessed by owner questionnaire. Nighttime activity was increased after ABT-116 treatment (*P* = 0.01). Kinetic gait analysis did not reveal significant treatment effects. Use of rescue treatment decreased with treatment in the ABT-116 and Carprofen groups (*P* < 0.001). Questionnaire score and activity count at the end of treatment were correlated with age, clinical severity at trial entry, and outcome measure baseline status (S_R_ ≥ ±0.40, *P* ≤ 0.005). Placebo treatment effects were evident with all variables studied.

**Conclusion:**

Treatment of hip OA in client-owned dogs is associated with a placebo effect for all variables that are commonly used for efficacy studies of analgesic drugs. This likely reflects caregiver bias or the phenomenon of regression to the mean. In the present study, outcome measures with significant effects also varied between groups, highlighting the value of using multiple outcome measures, as well as an a priori analysis of effect size associated with each measure. Effect size data from the present study could be used to inform design of future trials studying analgesic treatment of canine OA. Our results suggest that analgesic treatment with ABT-116 is not as effective as carprofen or tramadol for treatment of hip arthritis pain in client-owned dogs.

## Background

Osteoarthritis (OA) is one of the most common orthopaedic diseases of dogs. It is a chronic condition associated with progressive destruction of joint tissues, including bone, cartilage, and synovium. Estimates in one study suggest that up to 20% of the adult canine population has some type of OA [1]. OA is common in the weight-bearing joints of medium to large-sized dogs, although it may affect any synovial joint. Arthritis secondary to hip dysplasia is particularly common. The prevalence of hip dysplasia varies in different breeds and in many large breeds exceeds 50% [2]. Other appendicular joints that are commonly affected with OA in dogs include the stifle, the shoulder and the elbow. Age, conformation and extrinsic factors, such as diet and exercise, also have significant effects on OA phenotype in dogs [3-5]. Assessment of joint pain and mobility in a chronic disease, such as OA, is challenging in both human beings and dogs, since the disease changes slowly over time, flare-ups may occur [6], and medications may have relatively small treatment effects.

Current treatments for OA are palliative, with salvage surgical treatment, such as total joint replacement, as an option for treatment of end-stage disease. In human beings, opioid analgesics, non-steroidal anti-inflammatory drugs (NSAIDs), as well as injection of intra-articular medication, such as corticosteroids or hyaluronic acid, are widely used for the symptomatic treatment of OA [7]. At the present time, there are no disease modifying osteoarthritic drugs (DMOADs) approved for the treatment of human OA, although various classes of drugs have been studied, including matrix-metalloproteinase inhibitors, bisphosphonates, cytokine inhibitors, calcitonin, and nitric oxide synthase inhibitors [8]. In the United States, NSAIDs are widely used for management of canine OA.

Animal models of OA have been an important bridge between in-vitro studies and human clinical trials, and have been extensively used in research to further understanding of the etiopathogenesis of OA, identification of OA markers, and determination of therapeutic efficacy [9]. A variety of species have been used for model research, including mice, rats, rabbits, guinea pigs, and dogs. Models of both spontaneous and induced disease have been used [9]. Spontaneous disease models often provide a good model of OA pathology, but have the disadvantage of variation in time of onset and OA severity. Use of small laboratory animal models, such as mice, rats, or rabbits, for initial determination of safety and preliminary efficacy is common [9]. However, efficacy in such models may not yield similar results in higher species, such as dogs, or ultimately humans. A variety of canine OA models using both induced and naturally occurring arthritic disease have been used for studying efficacy of analgesics, DMOADs, and joint supplements [10-14].

In human clinical trials, placebo effects are well recognized [15,16]. A meta-analysis study of 198 human clinical trials on analgesic treatment of OA found that placebo treatment was effective in relieving pain, stiffness and self-reported function, compared to untreated controls [17]. The effect size (ES) for the placebo group was 0.51-0.55 in studies using a range of therapies including non-pharmacological, pharmacological, and surgical treatments. Such a phenomenon is not generally reproduced in model studies in experimental animals, but is a key feature of OA studies that use client-owned dogs. This is potentially valuable, as drugs that have a robust efficacy in a client-owned canine model may be more likely to yield an efficacious result in human clinical trials. One of the major challenges with trial studies in client-owned dogs is selection of appropriate outcome-measures that can effectively measure clinical improvement. Few trials have studied multiple outcome measures or reported effect sizes in detail. Such information can be very valuable by informing trial design and pre-trial power analysis of future randomized controlled efficacy studies.

In the current study, we present data describing multiple outcome measures of mobility in a randomized controlled trial studying naturally occurring hip OA in client-owned dogs. Individual measures have been previously used to study oral NSAID treatment effects in dogs, but not a combination of objective outcome measures and a validated client questionnaire in a single trial; studies of other classes of analgesic drugs are not well characterized. The overall goal of this work was to inform experimental trial design by determining effect sizes of key outcome-measures relevant to trial studies evaluating analgesic treatment of OA pain using client-owned dogs. We hypothesized that treatment effects associated with different outcome measures would vary when drugs with different modes of action are compared in a clinical trial. Our results suggest that multiple outcome measures and a placebo control group are needed to fully determine clinical efficacy in studies that evaluate client-owned dogs.

## Results

### Clinical details of the trial cohort

**Figure 1:**
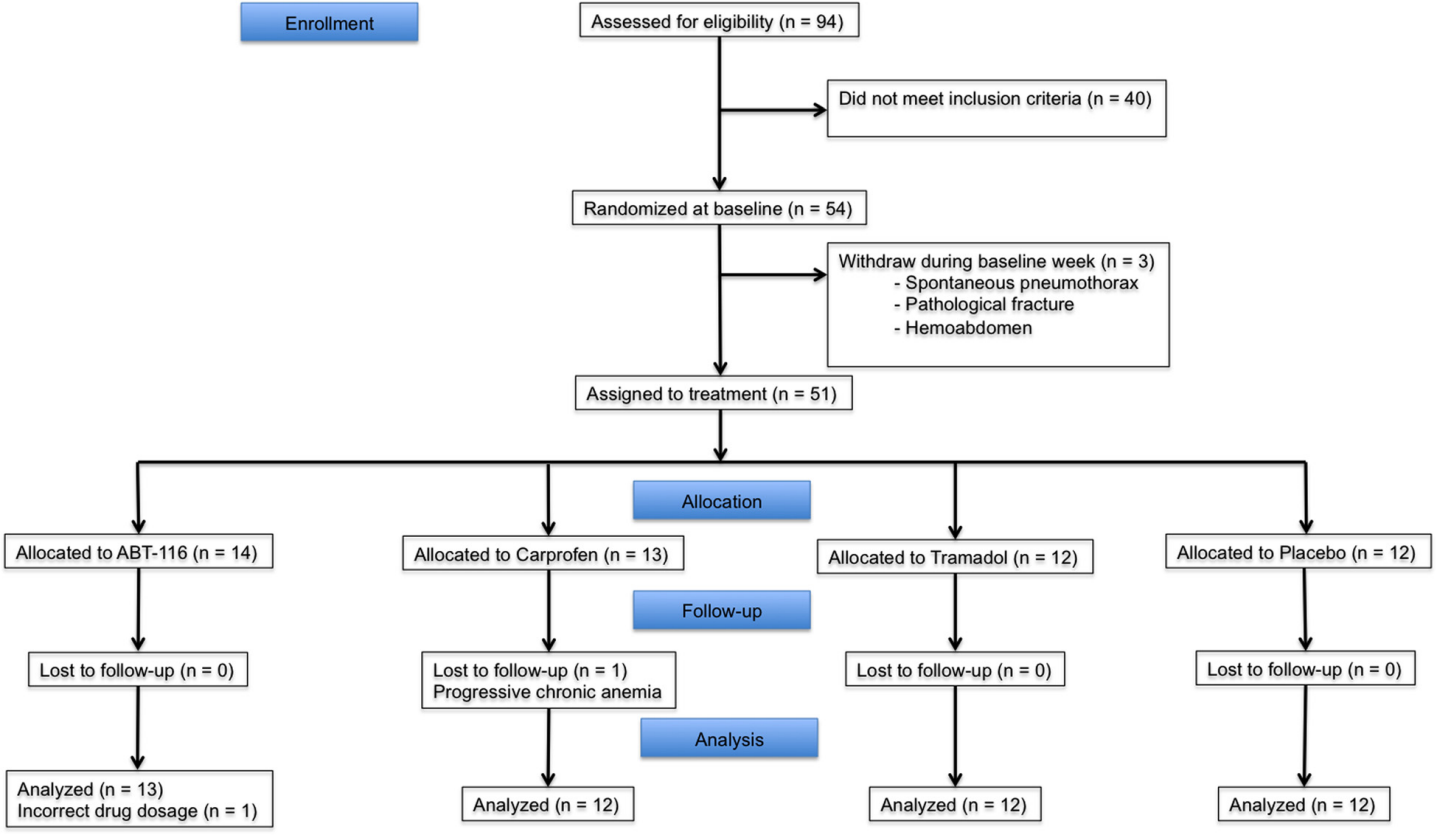
**Clinical trial flow diagram.** The diagram indicates loss of participants during enrollment, allocation, follow-up, and analysis.

Ninety-four client-owned dogs were evaluated at the University of Wisconsin-Madison Veterinary Medical Teaching Hospital (VMTH) between March 2010 and March 2011, in response to trial advertisements or referral by primary care veterinarians. A flow diagram of the clinical trial is presented in Figure 1. Fifty-four client-owned dogs met the inclusion criteria and were enrolled in the study. Forty dogs did not meet the inclusion criteria because of back pain associated with cauda equina syndrome (n = 16) and no hip OA, untreated cranial cruciate ligament rupture (n = 10), lack of OA in hip joints on radiographs (n = 9), lack of pain on manipulation of hip joints (n = 4), and medially luxating patella (n = 1). Five dogs were excluded from the study after enrollment for reasons unrelated to the trial. Four dogs were excluded for development of serious health problems: severe progressive anemia, spontaneous pneumothorax, hemoabdomen from a splenic mass, and development of pathological distal radial fracture because of osteosarcoma. The remaining dog was excluded because of an error in dosage of ABT-116 (transient receptor potential vanilloid 1, TRPV1 antagonist) during the trial. The remaining 49 enrolled dogs successfully completed the trial (Table 1). All dogs were affected with unilateral (n = 28) or bilateral (n = 21) hip pain based on clinical examination, and unilateral (n = 11) or bilateral (n = 38) hip OA radiographically. Seven dogs had received previous surgical treatment of orthopaedic conditions. Unilateral excision arthroplasty of the hip was performed in two dogs for traumatic hip luxation and hip dysplasia. Unilateral total hip replacement was performed in one dog due to severe hip arthritis. Two dogs were treated with bilateral stifle stabilization surgery for cranial cruciate ligament rupture. Treatment with a tibial plateau leveling osteotomy and an extracapsular stabilization was used in one dog. In the other dog, a tibial tuberosity advancement and extracapsular stabilization were used. The osteotomy sites in both dogs had completely healed and orthopaedic examination did not reveal any discomfort on manipulation of these stifles. The stifles with osteotomies were dynamically stable with no detectable cranial tibial thrust on physical examination. Stifles treated with the extracapsular suture technique were clinically stable when cranial drawer and tibial thrust tests were performed. Surgical removal of an osteochondral flap from the shoulder and removal of a fragmented medial coronoid process from the elbow joint had been previously performed in the other dogs.

**Table 1:** Baseline clinical characteristics for the ABT-116, carprofen, tramadol, and placebo treatment groups

	**ABT-116**	**Carprofen**	**Tramadol**	**Placebo**
	**(n = 13)**	**(n = 12)**	**(n = 12)**	**(n = 12)**
**Body weight (kg)**	34.9 ± 6.0	34.2 ± 6.9	34.9 ± 9.9	34.0 ± 8.4
**Age (years)**	6.0 ± 4.1	8.6 ± 3.6	9.2 ± 3.2	9.5 ± 4.2
**Gender**	SF – 8	SF – 6	SF – 7	SF – 8
	NM - 5	NM - 6	F - 1	NM – 3
			NM - 4	M - 1
**Clinical Severity**	2 (1, 3)	1.5 (1, 3)	2, (1, 3)	2 (1, 3)

**Note**: All values represent mean ± standard deviation, except for clinical severity, which is reported as median (range). n – sample number. SF – ovariohysterectomized female; F – female; NM – castrated male; M – male. At the start of the trial (Day 1), dogs were graded for clinical severity of lameness and pain (normal – 0; mild – 1; moderate −2; and severe - 3), and orthopaedic physical examination was performed.

Some of the 49 enrolled dogs were also affected with OA of other joints. These conditions included: spondylosis at the lumbosacral junction (n = 19), elbow arthritis (n = 5), shoulder arthritis (n = 2), and bilateral medial shoulder instability (n = 1). Other health conditions identified in the cohort included cystic calculi as an incidental finding (n = 2); superficial pyoderma treated with oral cephalexin during the trial (n = 2); allergic dermatitis (n = 2); urinary incontinence controlled with phenylpropanolamine (n = 1), cognitive dysfunction syndrome treated with selegiline (n = 1), and low-grade chronic bronchopneumonia (n = 1). There were 24 dogs that were receiving joint supplements orally (glucosamine, chondroitin sulfate) (n = 20), in specific diets (Prescription Diet® j/d® Canine Mobility, Hill’s Pet Nutrition, Inc.) (n = 4), or both (n = 2) at the time of trial entry.

Eighteen trial dogs with hip OA also had lumbosacral pain based on palpation of the lumbosacral region; 12 of these dogs had radiographic evidence of discospondylosis and arthritis at the lumbosacral junction. During the second and third visit days of the trial, 2 of these 12 dogs did not show any pain response upon repeated physical examination. There were no radiographic abnormalities noted in the other 6 dogs with lumbosacral pain clinically. At follow-up visits, 3 of these 6 dogs did not show any signs of pain upon repeated examination. There were also 7 dogs that had lumbosacral spondylosis and arthritis radiographically in which clinical signs of spinal pain were not detected.

The cohort of 49 dogs consisted of the following breeds: Labrador retriever (13), mixed breed (13), German shepherd (5), Rottweiler (5), Portuguese Water dog (3), Golden retriever (2), and one of each of the following breeds: Airedale terrier, Mastiff, Standard Poodle, American Bulldog, Hovawart, St. Bernard, Border collie, and German Short-haired pointer. There were 26 spayed females, 21 neutered males, one male, and one female. Body weight and age were 34.5 ± 7.7 kg and 8.3 ± 3.9 years respectively. There were 13 dogs in the ABT-116 group and 12 in the remaining 3 groups. There were no significant differences between groups in either body weight (*P* = 0.98) or age (*P* = 0.10). There were no significant differences in clinical severity of lameness between groups (*P* = 0.67). Median clinical severity scores (maximum score of 3) were ABT-116 - 2 (1, 3); Carprofen (non-steroidal anti-inflammatory drug NSAID) – 1.5 (1, 3); Tramadol (opiate) – 2 (1, 3); and Placebo – 2 (1, 3). No side effects associated with trial medication were detected during the study, other than increased rectal temperature in ABT-116 group. One dog developed bloody diarrhea, which resolved with medical management and was attributed to dietary indiscretion. This dog was removed from the trial until clinical signs had resolved and then completed the remaining trial period without complication.

### Measurement of plasma drug concentrations

Plasma concentrations of ABT-116, Carprofen, and Tramadol immediately before and 3 hours after dosing on the first and last days of the treatment period are presented in Table 2. One dog was excluded from each group because high plasma concentrations were detected in the Day 8 pre-treatment sample. In these dogs, the analyte was also not detected in the pre- (Carprofen and Tramadol groups) or post-treatment (ABT-116 group) Day 22 samples. On Day 8, the analyte was not detected in four post-treatment plasma samples (ABT-116 group n = 1, Carprofen group n = 1, Tramadol group n = 2). On Day 22, the analyte was not detected in four post-treatment plasma samples in the Tramadol group. In the ABT-116 group, plasma concentrations after dosing were significantly increased on Day 22, when compared with Day 8 (*P* < 0.001), whereas in the Tramadol group, plasma concentrations after dosing were significantly decreased in Day 22, when compared with Day 8 (*P* < 0.002). ABT-116, carprofen or tramadol were not detected in any of the plasma samples from dogs in the Placebo group.

**Table 2:** Plasma concentrations of ABT-116, carprofen, tramadol, and placebo before and after the first and last doses of the treatment period

**Treatment group**	**Trial day 8**			**Trial day 22**		
	**Before dosing**	**3 hours after dosing**	**n**	**Before dosing**	**3 hours after dosing**	**n**
**ABT-116**	0 ± 0	569 ± 294	12^#^	1,277 ± 644	1,589 ± 700***	12^#^
**ABT-116 metabolite**	0 ± 0	115 ± 63	11^#^	592 ± 256	538 ± 244***	11^#^
**Carprofen**	0 ± 0	3,386 ± 2,282	11^#^	3,752 ± 2,211	4,779 ± 2,789	11^#^
**Tramadol**	0.0 ± 0.0	39.3 ± 35.3	11^#^	0.6 ± 1.9	7.1 ± 8.8**	11^#^

**Note**: All values represent mean ± standard deviation in ng/ml. n – sample number. ^#^One dog in the treatment group was excluded because high drug concentrations were detected in the pre-treatment sample and low concentrations in one or more post-treatment samples. ABT-116, Carprofen, and Tramadol were not detected in any plasma sample from the Placebo group. Significance of changes in plasma drug concentrations from post-treatment sample on Day 8 is indicated as follows: ** - *P* < 0.01; *** - *P* < 0.001.

### Rectal temperature, heart rate, and respiration

**Figure 2:**
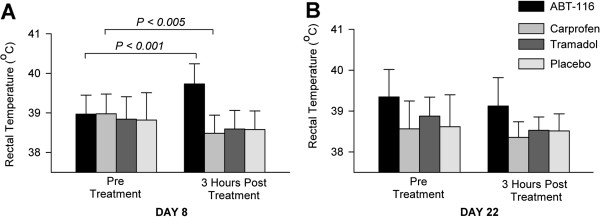
**Effect of ABT-116, carprofen, tramadol, and placebo treatment on rectal temperature in dogs with hip osteoarthritis.A**. Pre- and post-treatment rectal temperatures on the first day of medication (Day 8). **B**. Pre and post treatment rectal temperatures on last day of medication (Day 22).

Overall, there were significant treatment effects on rectal temperature in the study. In the ABT-116 group, there was an increase in rectal temperature after the initial dose of medication on Day 8 (*P* < 0.001), whereas rectal temperature decreased after treatment in the Carprofen group (*P* < 0.005) (Figure 2). In the Tramadol and Placebo groups, there were no significant treatment effects on rectal temperature. On Day 22 of the trial, when the last dose of medication was given, there were no acute treatment effects on rectal temperature in any group (*P* > 0.07). However, in the ABT-116 group, baseline temperature was increased compared with Day 1 (*P* < 0.05), but not Day 8 (*P* = 0.75). ES for rectal temperature in all treatment groups for Days 8 and 22 are presented in Table 3.

**Table 3:** Effect sizes and confidence intervals for rectal temperature treatment effects after administration of the first and last doses of trial medication

**Treatment group**	**Rectal temperature**			
	**Day 8**		**Day 22**	
	**ES**	**95% CI**	**ES**	**95% CI**
**ABT-116**	1.4	0.54 to 2.26	−0.29	−1.06 to 0.49
**Carprofen**	−1.00	−1.85 to −0.15	−0.35	−1.16 to 0.46
**Tramadol**	−0.36	−1.17 to 0.45	−0.97	−1.82 to −0.12
**Placebo**	−0.33	−1.13 to 0.48	−0.16	−0.96 to 0.64

**Note**: ES – effect size; CI – confidence interval.

The median and range of heart rate for dogs on Day 1 was 120 (68, 200) beats per minute and respiratory rate was 20 (16, 50) breaths per minute. Most dogs (42/49) were panting during the examination. There were no differences in heart rate between groups (*P* > 0.05).

### Hip range-of-motion and thigh circumference

Hip range of motion (ROM) in the worst affected pelvic limb was decreased when compared with the contralateral pelvic limb (*P* < 0.005) in the Tramadol group at trial entry. In the other three groups, hip ROM was not significantly different between pelvic limbs. Additionally, hip ROM was decreased in the ABT-116 and Tramadol groups, when compared with the Placebo group (*P* < 0.05) at trial entry. There were no changes in hip ROM in either the worst affected pelvic limb, or the contralateral pelvic limb with treatment in any group (*P* > 0.05). ES for treatment effect on hip ROM in the more severely affected hip joint in the Placebo group was 0.13 (95% CI - 0.68 to 0.93).

Hip ROM at the end of trial was correlated with the mean baseline value (S_R_ > 0.58, *P* < 0.001) for both the worst affected pelvic limb and the contralateral limb. Correlations with body weight, age, and clinical severity at trial entry were not significant.

Thigh circumference was decreased in the worst affected pelvic limb, when compared with the contralateral limb (*P* < 0.01) in the ABT-116 and Tramadol groups at trial entry, but not the Carprofen and Placebo groups. There were no changes in thigh circumference in either the worse affected pelvic limb, or the contralateral pelvic limb with treatment in any group (*P* > 0.05). ES for treatment effect on thigh circumference in the worst affected hip joint in the Placebo group was 0.2 (95% CI - 0.60 to 1).

Thigh circumference at the end of the trial was inversely correlated with age (S_R_ > −0.68, *P* < 0.001) and clinical severity at trial entry (S_R_ > −0.30, *P* < 0.05) for both the worst affected pelvic limb and the contralateral limb. In addition, thigh circumference at the end of the trial was correlated with mean baseline (S_R_ > 0.85, *P* < 0.001).

### Canine brief pain inventory questionnaire

Results are summarized in Table 4. Overall, there were no significant changes in total canine brief pain inventory (CBPI) score (*P* = 0.21), pain severity score (*P* = 0.42) or pain interference score (*P* = 0.11) between treatment groups. For total score, improvements in response to treatment were found in the Carprofen (*P* < 0.005) and Tramadol (*P* < 0.001) groups, but not ABT-116 (*P* = 0.09) or Placebo (*P* = 0.30) groups. For pain severity scoring, improvements in response to treatment were found in the Carprofen (*P* = 0.005) and Tramadol (*P* < 0.05) groups, but not ABT-116 (*P* = 0.24) or Placebo (*P* < 0.27) groups. For pain interference scoring, changes in response to treatment were also found for Carprofen (*P* < 0.005), and Tramadol (*P* < 0.001) groups, but not ABT-116 (*P* = 0.13), or Placebo (*P* = 0.35) groups. Improvements in pain interference scoring after tramadol treatment were most evident for ability to rise to standing after lying down and ability to climb stairs or curbs. ES for total score, pain score, and pain interference scores for each group are presented in Table 5.

**Table 4:** Effect of analgesic treatment on canine brief pain inventory (CBPI) questionnaire scores

	**ABT-116 (n = 13)**	**Carprofen (n = 12)**	**Tramadol (n = 12)**	**Placebo (n = 12)**
**Total CBPI Score**	−20.3 ± 39.5	−40.6 ± 34.9**	−38.5 ± 30.0***	−13.5 ± 42.7
Statistical Power	0.07	0.37	0.36	
Sample Size for Power > 0.8	n = 580	n = 34	n = 35	
**Pain Severity Score**	−15.3 ± 45.0	−39.4 ± 34.5**	−24.9 ± 39.0	−14.7 ± 43.7
Statistical Power	0.05	0.32	0.19	
Sample Size for Power > 0.8	n > 1,000	n = 41	n = 260	
**Pain Interference Score**	−20.1 ± 44.8	−41.8 ± 37.3**	−47.2 ± 29.2***	−12.6 ± 44.4
Statistical Power	0.07	0.39	0.60	
Sample Size for Power > 0.8	n = 500	n = 32	n = 19	

**Note**: Data represent percentage change in Week 3 of the trial, compared with mean baseline. Power analysis results compare each treatment group with placebo. Significance of changes in questionnaire scoring from mean baseline to end of the trial is indicated as follows: ** - *P* ≤ 0.01; *** - *P* ≤ 0.001.

**Table 5:** Effect sizes and confidence intervals for treatment effects on canine brief pain inventory (CBPI) score

**Treatment group**	**Total CBPI score**		**Pain severity score**		**Pain interference score**	
	**ES**	**95% CI**	**ES**	**95% CI**	**ES**	**95% CI**
**ABT-116**	−0.33	−1.1 to 0.45	−0.31	−1.08 to 0.47	−0.30	−1.07 to 0.48
**Carprofen**	−0.64	−1.46 to 0.18	−0.55	−1.36 to 0.27	−0.66	−1.48 to 0.17
**Tramadol**	−1.01	−1.86 to −0.16	−0.55	−1.37 to 0.26	−1.30	−2.18 to −0.42
**Placebo**	−0.34	−1.14 to 0.47	−0.33	−1.14 to 0.47	−0.35	−1.52 to 0.46

**Note**: ES – effect size; CI – confidence interval.

Overall, total CBPI score at the end of the trial was correlated with age (S_R_ = 0.49, *P* < 0.001), clinical severity at trial entry (S_R_ = 0.41, *P* < 0.005), and mean baseline score (S_R_ = 0.71, *P* < 0.001). Similar results were obtained when pain and pain interference scores were analyzed. However, within groups there were no significant correlations between weight, gender, age, or clinical severity and percentage changes in total score, pain severity score or pain interference score.

The pattern of questionnaire responses for the four questions describing pain was similar. The best discrimination between no pain and extreme pain was seen for the questions rating pain at its worst and average pain. The pattern of scoring for adjacent pain score values was not closely clustered. Similarly, the pattern of scoring for distant pain score values did not show wide separation. Similar results were found when the pattern of scoring for the six questions describing pain interference was examined. Large differences in the pattern of owner responses to questionnaires in different treatment groups were not observed.

### Accelerometer measurement of dog activity

Results are summarized in Table 6. Activity data were available for 48 dogs. A monitor malfunction occurred in one dog. In the Tramadol group, one dog was excluded from nighttime activity analysis because the owner mistakenly removed the accelerometer for most of this time period during the trial. There was a wide range in the daily activity of individual dogs. In all groups, the most active dogs accumulated several times the number of daily accelerations recorded by the least active dog (ABT-116 group – 5.8 fold; Carprofen group – 13.9 fold; Tramadol group – 6.2 fold; Placebo group – 4.7 fold).

**Table 6:** Effect of analgesic treatment on dog activity measured using an accelerometer

	**ABT-116 (n=13)**	**Carprofen (n=11)**	**Tramadol (n=12)**	**Placebo (n=12)**
**Total activity**	9.7 ± 24.2	2.7 ± 9.9	−6.6 (−22.5, 36.9)^**a**^	−1.2 ± 18.4
Statistical Power	0.24	0.10		
Sample Size for Power > 0.8	n = 59	n = 204		
**Daytime activity**	5.8 ± 25.2	5.5 ± 11.3	−4.9 (−22.6, 37.8)^**a**^	−0.5 ± 18.1
Statistical Power	0.11	0.16		
Sample Size for Power > 0.8	n = 185	n = 93		
**Nighttime activity**	58.4 ± 97.4**	−23.4 ± 23.8**	3.0 ± 38.7^**b**^	−4.3 ± 44.8
Statistical Power	0.84	0.3	0.09	
Sample Size for Power > 0.8	N/A	n = 41	n = 275	

**Note**: Daytime activity - 6 am to 10 pm; Nighttime activity - 10 pm to 6 am. Data represent percentage change in Week 3 of the trial, compared with the baseline week. Power analysis results compare each treatment group with placebo. Significance of changes in activity from baseline to end of the trial is indicated as follows: ** - *P* ≤ 0.01**.**^**a**^Data for Total activity and Daytime activity in the Tramadol group were not normally distributed. ^**b**^One dog in Tramadol group was excluded from nighttime activity analysis because the owner removed the accelerometer during this period, so n = 11 in this cell.

Total daily activity and daytime activity in Week 3 during the second week of treatment were higher than in the baseline week after treatment in the ABT-116 and Carprofen groups, but not the Tramadol or Placebo groups. There were no significant changes in total activity or daytime activity after treatment in any group, although higher daytime activity was most evident after treatment in the Carprofen group (*P* = 0.14). During the nighttime period, activity was higher after treatment in the ABT-116 and Tramadol groups, but not the Carprofen and Placebo groups. Change in activity during the nighttime period was increased with ABT-116 treatment compared with the Carprofen group (*P* = 0.01) and higher compared with the Tramadol (*P* = 0.13) and Placebo groups (*P* = 0.06). With ABT-116 treatment, nighttime activity was increased (*P* = 0.01), and with carprofen treatment activity was decreased (*P* = 0.01). ES for total activity, daytime activity, and nighttime activity for all groups are presented in Table 7.

**Table 7:** Effect sizes and confidence intervals for treatment effects on dog activity

**Treatment group**	**Total activity**		**Daytime activity**		**Nighttime activity**	
	**ES**	**95% CI**	**ES**	**95% CI**	**ES**	**95% CI**
**ABT-116**	0.19	−0.59 to 0.96	0.13	−0.64 to 0.90	0.51	−0.30 to 1.32
**Carprofen**	−0.02	−0.82 to 0.78	0.01	−0.79 to 0.81	−0.53	−1.34 to 0.29
Tramadol*	−0.1		−0.1		0.07	−0.77 to 0.90
**Placebo**	−0.12	−0.92 to 0.68	−0.10	−0.90 to 0.70	−0.19	−0.99 to 0.62

**Note**: ES – effect size; CI – confidence interval. *The data in the Tramadol group only had normal distribution for nighttime activity data. Effect size for total activity and daytime activity in the Tramadol group was calculated using the Cliff’s Delta statistic.

Total activity at the end of the trial was inversely correlated with age (S_R_ = −0.64, *P* < 0.001) and clinical severity at trial entry (S_R_ = −0.40, *P* = 0.005). End-of-trial activity was also highly correlated with baseline activity (S_R_ = 0.93, *P* < 0.001). Similar results were obtained when daytime and nighttime activities were analyzed separately. Treatment-associated changes in total activity were significantly influenced by gender in the ABT-116 (S_R_ = −0.76, *P* < 0.005) and Tramadol (S_R_ = −0.88, *P* < 0.001) groups, but not the Carprofen or Placebo groups. Activity was higher in males after treatment in the ABT-116 and Tramadol groups, relative to female dogs.

### Force-plate analysis-of-gait

Force-plate analysis data was available in 41 dogs. The remaining 8 dogs were excluded due to use of rescue medication within 48 hours before examination, or the presence of a gait abnormality that impeded data collection. Results are summarized in Table 8.

**Table 8:** Effect of analgesic treatment on gait kinetics measured using a force-plate

**Kinetic parameters**	**ABT-116 (n = 12)**	**Carprofen (n = 11)**	**Tramadol (n = 9)**	**Placebo (n = 9)**
**Peak Vertical Force**	4.4 ± 6.8	1.2 ± 7.5	2.1 ± 4.9	2.8 ± 10.6
Statistical Power	0.07	0.07	0.05	
Sample Size for Power > 0.8	485	445	>1000	
**Peak Vertical Impulse**	2.3 ± 13.4	2.4 ± 7.1	−1.1 ± 6.9	−1.8 ± 6.9
Statistical Power	0.14	0.24	0.05	
Sample Size for Power > 0.8	100	45	>1000	
**Falling Slope**	−3.2 ± 19.0	−0.6 ± 16.2	4.6 ± 19.5^a^	11.1 ± 35.2^a^
Statistical Power	0.10	0.14	0.08	
Sample Size for Power > 0.8	185	95	275	

**Note**: Data represent percentage change in Week 3 of the trial, when compared with mean baseline. Power analysis results compare each treatment group with placebo. ^a^Falling slope is more negative after treatment (undesirable effect).

Changes in peak vertical force (F_z_), vertical impulse (VI), and falling slope (FS) with treatment were not significantly different between groups (*P* > 0.05). Higher F_z_ values were recorded in all groups on Day 22, when compared with mean baseline. However, F_z_ after treatment in the ABT-116 (*P* = 0.05), Carprofen (*P* = 0.6), Tramadol (*P* = 0.23), and Placebo (*P* = 0.45) groups was not significantly different from mean baseline. Higher vertical impulses (VIs) were recorded after treatment in the ABT-116 and Carprofen groups, but not the Tramadol or Placebo groups. There were no significant differences in VI after treatment in any group (*P* ≥ 0.28). Falling slope (FS) values recorded after treatment in the Tramadol and Placebo groups had a larger negative value (dogs were unloading their limb faster). Small reductions in the FS value were noted in the ABT-116 and Carprofen groups (slower unloading). However, there were no significant differences in FS after treatment in any group (*P* ≥ 0.37). The ES for F_z_, VI, and FS are presented in Table 9.

**Table 9:** Effect sizes and confidence intervals for treatment effects on kinetic gait analysis

**Treatment group**	**Peak vertical force (F**_**z**_**)**		**Peak vertical impulse (VI)**		**Falling slope (FS)**	
	**ES**	**95% CI**	**ES**	**95% CI**	**ES**	**95% CI**
**ABT-116**	0.11	−0.68 to 0.90	0.12	−0.67 to 0.90	0	−0.79 to 0.79
**Carprofen**	0.06	−0.78 to 0.90	0.21	−0.63 to 1.05	0.06	−0.78 to 0.89
**Tramadol**	0.07	−0.83 to 0.97	−0.09	−0.97 to 0.80	−0.3	−1.19 to 0.59
**Placebo**	−0.07	−0.97 to 0.83	−0.31	−1.22 to 0.59	−0.05	−0.95 to 0.85

**Note**: ES – effect size; CI – confidence interval.

Kinetic analyses at the end of the trial were significantly related to mean baseline (S_R_ > 0.80, *P* < 0.001). In the Carprofen group, percentage change in F_z_ and FS with treatment was significantly correlated with age (S_R_ > 0.68, *P* < 0.05). Percentage change in FS with treatment was also correlated with gender (S_R_ = 0.78, *P* < 0.005) and clinical severity (S_R_ = 0.64, *P* < 0.05). FS response to treatment was greater in males versus females. In the Tramadol group, percentage change in F_z_ with treatment was inversely correlated with clinical severity (S_R_ = −0.77, *P* < 0.05). No significant correlations were identified with VI.

### Use of rescue medication

**Figure 3:**
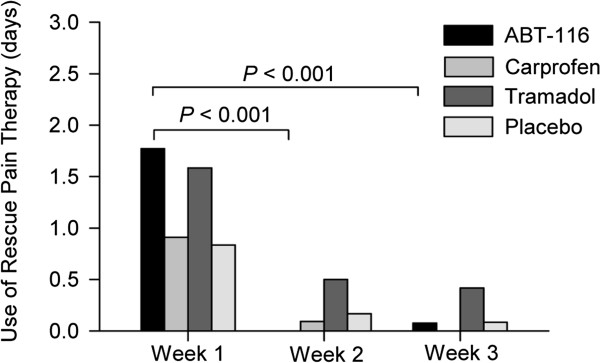
**Effect of ABT-116, carprofen, tramadol, and placebo treatment on use of rescue medication in Weeks 1, 2 and 3 of the trial.** No significant difference was noted between Week 2 and 3 of treatment in any group.

Overall, change in use of rescue treatment in each therapeutic group was not significantly different (*P* > 0.05). Overall, there was a reduction in the number of days that rescue medication was used during the trial period in the ABT-116 (*P* < 0.001), Carprofen (*P* < 0.001), and Placebo (*P* < 0.01) groups, but not the Tramadol group (*P* > 0.19). Significant changes in use of rescue medication between each week of the trial are summarized in Figure 3. When number of doses of rescue medication used in each trial week was analyzed, similar results were obtained. The ES values in the ABT-116, Carprofen, Tramadol and Placebo groups for reduction in use of rescue medication after treatment were −0.6, -0.7, -0.4 and −0.3 respectively.

Use of rescue medication in Week 3 was not significantly correlated with weight, gender, age, clinical severity, or use of rescue medication during the baseline week. In the ABT-116 group, reduction from baseline in use of rescue medication in Week 3 of the trial was inversely correlated with clinical severity (S_R_ = −0.56, *P* < 0.05). No significant correlations with treatment responses were identified for the Carprofen, Tramadol, or Placebo groups.

### Relationships between outcome measures of mobility

Relationships between outcome measures at baseline are summarized in Table 10. Baseline CBPI total score was inversely correlated with baseline daily activity (S_R_ = −0.35, *P* < 0.05), and mean baseline F_z_ (S_R_ = −0.42, *P* < 0.01), and positively correlated with baseline use of rescue medication (S_R_ = 0.37, *P* < 0.01). Baseline daily activity was inversely correlated with use of rescue medication (S_R_ = −0.32, *P* < 0.05).

**Table 10:** Correlations between baseline measures of dog mobility

**Outcome measure**	**Total CBPI**		**Total activity**		**Peak vertical force (F**_**z**_**)**		**Use of rescue medication**	
	**S**_**R**_	***P *value**	**S**_**R**_	***P *value**	**S**_**R**_	***P *value**	**S**_**R**_	***P *value**
**Total CBPI**			−0.35	0.02	−0.42	0.006	0.37	0.009
**Total Activity**	−0.35	0.02			0.14	NS	−0.32	0.03
**Peak Vertical Force (F**_**z**_**)**	−0.42	0.006	0.14	NS			−0.21	NS
**Use of rescue medication**	0.37	0.009	−0.32	0.03	−0.21	NS		

**Note:** CBPI – Canine brief pain inventory questionnaire. Correlations used baseline or mean baseline data as applicable. Significant Spearman rank order correlations (S_R_) results are presented when *P* < 0.05. NS – not significant.

A summary of treatment effects is presented in Table 11. In the ABT-116 group, percentage changes in dog activity and F_z_ with treatment were significantly and inversely correlated (S_R_ = −0.65, *P* < 0.05). In the Placebo group, percentage change in dog activity and change in use of rescue treatment were significantly and inversely correlated (S_R_ = −0.58, *P* < 0.05). No significant treatment effect correlations were identified in the Carprofen and Tramadol groups.

**Table 11:** Summary of ABT-116, carprofen, tramadol, and placebo treatment effects on dog mobility

**Outcome measure**	**ABT-116**		**Carprofen**		**Tramadol**		**Placebo**	
	**Significance**	**ES**	**Significance**	**ES**	**Significance**	**ES**	**Significance**	**ES**
**CBPI questionnaire**								
Total CBPI score	NS	**	*P* < 0.005	**	*P* = 0.001	***	NS	**
Pain Severity Score	NS	**	*P* < 0.005	**	NS	**	NS	**
Pain Interference Score	NS	**	*P* < 0.005	**	*P* < 0.001	***	NS	**
**Activity Monitoring**								
Total Activity	NS	*	NS	*	NS	*^a^	NS	*^a^
Daytime Activity	NS	*	NS	*	NS	*^a^	NS	*^a^
Nighttime Activity	*P* ≤ 0.01	**	*P* ≤ 0.01	**^a^	NS	*	NS	*^a^
**Kinetic Gait Analysis**								
Peak Vertical Force	NS	*	NS	*	NS	*	NS	*^b^
Vertical Impulse	NS	*	NS	**	NS	*^b^	NS	**^b^
Falling Slope	NS	*	NS	*	NS	**^c^	NS	*^c^
**Reduction in Use of Rescue Medication**								
	*P* < 0.001	**	*P* < 0.001	**	NS	**	NS	**

**Note:** ES – effect size; CBPI – canine brief pain inventory questionnaire. ES magnitude is indicated by * - Small, ES ≤ 0.2; ** - Medium, ES = > 0.2 and < 0.8; *** - Large, ES ≥ 0.8. ^a^ES indicating reduction in dog activity. ^b^ES indicating reduction in peak vertical force or peak vertical impulse values. ^c^ES indicating falling slope is more negative after treatment (undesirable effect). NS – not significant.

## Discussion

In the present study, we have analyzed four key clinical trial outcome measures (client-owned questionnaire, activity monitoring, measurement of ground reaction forces, and use of rescue treatment) in a single randomized controlled trial evaluating analgesic treatment of naturally occurring OA in dogs. Previous trial studies have often compared a NSAID, such as carprofen, with a placebo arm using a more limited range of outcome measures. Limited data are available directly comparing treatment effects of several different types of oral analgesics within a single trial. Placebo effects were identified for all outcome measures studied. All of the trial medications had relatively small treatment effects on outcome measures in dogs with moderate to severe hip arthritis, suggesting that development of new analgesic medications with greater clinical efficacy is needed. However, a relatively short treatment period was used in the present study. Additionally, we did not use a crossover study design. This would have minimized background variance, but increased the risk of subject dropout from a clinical trial with four treatment arms. Over time, incremental refinement in trial design will likely aid robust examination of treatment effects in client-owned dogs with arthritis.

The signalment and clinical signs of the dogs enrolled in the trial were typical for client-owned dogs with hip OA [14,18]. Many canine OA treatment trials have studied patients affected with a range of different arthritis conditions. In the present study, we used more restrictive selection criteria to minimize variation in the study population. There is evidence to suggest that OA of joints other than the hip in dogs, such as the elbow, may be more refractory to medical treatment [19]. Additionally, stifle OA in dogs is often associated with joint instability from cruciate rupture, which our clinical experience suggests is also more refractory to medical treatment. OA commonly affects multiple joints in the dog [20], and many dogs in the present study had multiple joints affected with OA, including degenerative changes to the lumbosacral spine. Therefore, some dogs may have had a neuropathic pain component to their clinical signs, although degenerative changes to the lumbosacral spine seen radiographically are not highly correlated with clinical signs [21].

In general, owner compliance with the trial was good. After group assignment, there were no significant differences in dog mobility between groups. Withdrawal from the trial after enrollment only occurred because of the presence of a complicating major health problem that was not attributable to trial medication, or because of a dosing error in one dog. After two weeks of treatment with ABT-116, plasma concentrations were significantly increased, suggesting that a loading phenomenon exists with oral dosing, with increased bioavailability from chronic dosing. In contrast, plasma concentrations of tramadol were significantly decreased after two weeks of treatment. This likely reflects altered drug metabolism, which is a complex phenomenon [22]. Despite low plasma concentrations of tramadol and its active metabolite after oral dosing, analgesic efficacy is still detectable experimentally [22]. The antinociceptive action of tramadol in the dog is not fully understood and may involve effects on α2-adrenoceptors, as well as inhibition of norepinephrine reuptake and μ-receptor agonism [22]. We felt the inclusion of a tramadol treatment group in this trial was important in order to provide data for future non-NSAID drug candidate testing.

Dosing with ABT-116 was associated with an acute elevation in rectal temperature. This is an established phenomenon associated with administration of TRPV1 antagonists in animal models [23-26], and is a biomarker of dosing efficacy. Attenuation of this side effect after continued treatment has been previously reported [23], and was also apparent in the ABT-116 group at recheck on Day 22. The hyperthermia associated with TRPV1 antagonists is related to their potency for blockade of receptor activation by protons [27]. We did not detect any clinical signs associated with this temporary side effect in the dogs of the present study. In human beings, there is individual variation in the degree of hyperthermia that develops with treatment, and there was also some variation in the small cohort of dogs in the present study. We also detected a small but significant acute decrease in rectal temperature after carprofen administration, although the biological significance of this observation is unclear.

Goniometry measurement indicated that hip ROM in the ABT-116 and Tramadol groups was decreased, when compared with the Placebo group. In the ABT-116 and Tramadol groups, thigh muscle mass in the worst affected limb was also decreased relative to the contralateral pelvic limb. However, there were no significant differences in overall mobility between groups based on clinical severity scoring at trial entry and no specific treatment effects on either hip ROM and thigh muscle mass. Improvement in these parameters would likely only occur over a treatment period longer than 2 weeks. Clinical response to long-term OA treatment with oral analgesics, such as carprofen, can continue for at least 12 weeks [28]. In the Placebo group, effect sizes for placebo treatment on hip ROM and thigh muscle mass were small (≤ 0.2).

The CBPI questionnaire is a validated reliable outcome measure for studies of OA in dogs [29]. In the present study, improvements in CBPI scores were seen in all treatment groups, including the Placebo group. The greatest improvement in CBPI score was seen in the Carprofen and Tramadol groups; significant improvements in both pain severity and pain interference scores were only seen in the Carprofen group. However, these improvements were not large enough to yield significant differences between groups. ES in the ABT-116 group was similar to the Placebo group. The magnitude of improvement seen with carprofen and placebo treatment was similar to other treatment studies using this outcome measure [29]. Interestingly, with tramadol treatment, improvement in pain interference was much greater than improvement in pain severity. Clinical improvements after tramadol were most evident in rising from lying down or ability to climb. This is interesting, particularly as plasma concentrations were decreased at Day 22, relative to Day 8. Additional studies are needed to assess in more detail the mode of action, metabolism, and clinical efficacy of tramadol in the dog during chronic oral dosing, as only sparse pharmacokinetic data are available to date [22].

ES of placebo treatment on CBPI scores was modest and did not represent a significant change. This effect likely represents a combination of caregiver placebo effect and the regression to the mean phenomenon [30]. Regression to the mean is a statistical phenomenon that occurs in repeated-measures studies. Because outcome variables are observed with random error around a true mean, relatively high or low observations are likely to be followed by less extreme ones nearer to the true mean [30]. In our study, regression to the mean was reduced by use of two baseline measurements and random allocation of subjects to treatment groups. We did not provide training on recognition of clinical signs of OA pain in dogs for participating owners. Poor face validity may limit the value of the questionnaire instrument to some extent [31]. This problem could be overcome in future work by altering the study design and training the owners by administering and then withdrawing a positive control such as carprofen [31]. This concern was also emphasized by the pattern of responses identified during correspondence analysis of the CPBI data. The pattern of responses in the four treatment arms of the study was fairly similar, suggesting that none of the treatments used had a major effect on owner perception of patient mobility. In addition, when the pattern of responses to individual questions was examined, clustering of divergent response options was seen, suggesting that owners were not able to consistently pain score the subject dogs within the context of the present study design.

Activity monitoring using an accelerometer is a reliable outcome measure for OA treatment studies in client-owned dogs [32]. However, in the present study we found that dog mobility was highly variable between individual dogs, with approximately a 5-fold difference or more between the least active dog and the most active dog in each treatment group. Treatment effects did not yield significant between-group differences in overall daily activity. The ABT-116 group was the only group in which dogs exhibited a significant increase in activity after treatment. This increase was identified during the nighttime period. In contrast, dogs that received carprofen treatment were significantly less active at night, but had higher activity in the daytime. Since client-owned dogs usually mirror the habitual activity of their owners, it would be expected that most dogs are more active in the daytime and less active at nighttime. Whether nighttime increases in activity represent a beneficial treatment effect is unclear. It may be that higher nighttime activity is due to increased discomfort, such that the dog is not able to get comfortable and sleep soundly. However, ABT-116 dogs also had improved CBPI scores after treatment and no decrease in daytime activity, so this increase in nighttime activity may simply reflect improved overall mobility. Further work is needed to more comprehensively understand treatment-induced changes in habitual activity in dogs with OA, particularly with regard to sleeping at nighttime. Overall, ES of treatment on daytime and total activity were small, and the large variation in habitual activity between individual dogs is a major factor contributing to small treatment effects in this study, probably acting to increase the magnitude of regression to the mean.

In general, larger F_z_ and VI values at both the walk and trot are associated with improved mobility in dogs [33-35]. Gait analysis at the trot provides a more sensitive indicator of lameness than analysis at the walk, particularly for mild lameness [36]. However, analysis at the walk may be better suited for dogs with more severe lameness, such as cranial cruciate ligament rupture [33,36]. In the present study, significant treatment effects on ground reaction forces were not identified, principally because of extensive variation between individual dogs. Our power analysis suggests that much larger sample sizes would be needed to detect significant drug treatment effects. We found that ES associated with treatment were greatest in the ABT-116 and Carprofen groups with improvement in kinetic gait analysis values, whereas ES associated with tramadol and placebo treatment were smaller and not generally associated with improvement in mobility. The placebo effect in force-plate gait analysis has been shown in other studies [14,34], and is again likely a consequence of the regression to the mean phenomenon.

In general, outcome measures, including hip ROM, thigh muscle mass, total CBPI, total daily activity, and kinetic gait analysis parameters at the end of the trial were significantly correlated with baseline measurements. This indicates reasonable measurement precision error over time. Although a two-week treatment period has often been used for this type of trial [14,19,29,32], longer treatment periods [28,37] may have led to larger improvements in dog mobility.

Analgesic treatment had significant effects on use of rescue medication by owners during the treatment period. This effect was most evident for ABT-116 and least evident for tramadol. One possible explanation for this finding in the tramadol group is that owners may have interpreted sedative effects from tramadol treatment as a sign of pain. This reinforces the idea that face validity of CBPI scoring may be improved by development of an a priori training protocol for owners as part of trial study design.

Thigh muscle mass, but not hip ROM, was also inversely correlated with dog age and clinical severity at trial entry, suggesting that aging and more severe impairment of mobility are associated with greater disuse atrophy of muscle in affected limbs. Similarly, total CBPI score at the end of the trial was also correlated with clinical severity at trial entry. This observation suggests the CBPI questionnaire may have reasonable face validity [38]. Dog activity at the end of the trial was also significantly and inversely correlated with clinical severity and dog age. Similar correlations were less evident for kinetic gait analysis parameters. Interestingly, use of rescue treatment in Week 3 was not correlated with clinical severity or use of rescue treatment in the baseline week. Whilst the ABT-116 ES for reduction in use of rescue treatment was larger than for tramadol, this difference was not reflected in CBPI questionnaire scoring. These observations suggest that the clinical criteria that owners used to decide whether or not use of rescue treatment was indicated were different from the criteria owners used for CBPI scoring of their dog.

There were several limitations with this study. A strength of this report is the use of client-owned dogs as the study population. However, this is also a limitation, as studies of naturally occurring OA in dogs are associated with factors that may be confounding, such as the presence of concurrent medical conditions, variations in the home environment of each dog, and the potential for owner errors in trial compliance. Randomization of trial participants is an important strategy to reduce bias and minimize the magnitude of regression to the mean [30]. In order to ensure equivalence of clinical severity in each arm of the study, we adjusted group assignment during recruitment of the last 12 study dogs to ensure equivalent clinical severity in each group. Alternate strategies during recruitment and treatment assignment, such as randomization blocked on clinical severity, could have been used to address this problem.

Measurement of plasma drug concentrations suggested that dosing or plasma sample labeling errors had occurred in a small number of samples. In addition, one dog was withdrawn after trial completion because of a dosing error. Owners were asked to maintain their habitual routine during the clinical trial period. However, some owners commented that they would allow their dog to become more active if they thought there was a clinical response to analgesic treatment. Therefore, owner bias may still influence outcome measures, even in a controlled trial. Our study was also restricted to use of medium to large breed dogs to enable kinetic gait analysis with the OR6-6-1000 force platform. Use of a pressure mat walkway would have allowed multiple footfalls to be collected in one pass, as well as reducing restrictions on the size of participating dogs [39]. Collection of 5 valid trials is often used during kinetic gait analysis in dogs [34,37]. Although trial repetition is an important factor contributing to variance, vertical forces are not significantly affected by trial repetition [40]. In some dogs in the present trial it was not possible to collect 5 valid trials for each limb, because of the severity of lameness. We analyzed kinetic gait data from the most severely affected pelvic limb. Other approaches could have been considered, such as calculation of a symmetry index [36].

The overall goal of this study was to determine ES of different outcome measures in a client-owned dog model. Many of the outcome measures we assessed had low statistical power, and a larger number of dogs/group would be required for significant results in an efficacy clinical trial. Use of a crossover study design would help to address this concern by better accounting for intra-individual variation between days and weeks of the trial. However, with a study that includes four treatment arms, use of crossover trial with four treatment and three washout periods would likely have reduced client compliance and increased the risk of dropout from the trial. The dosage of ABT-116 used was based on prior research. It is possible that altered dosage and duration of treatment may improve efficacy in treatment of OA pain. Dosage at 3 mg/kg three times daily resulted in effective TRPV1 inhibition based on the development of acute hyperthermia in treated dogs.

## Conclusions

Clinical trial studies of OA treatment in client-owned dogs have several experimental advantages over other in-vitro and in-vivo models. Firstly, naturally occurring disease models more closely OA of weight-bearing joints in human beings. Background variance in such canine trials also models common challenges involved in human clinical trials, including recruitment, randomization, participant compliance with trial design, and the potential for other confounding factors, such as other health problems or use of other medications. In addition, test compounds have to overcome a care-giver placebo effect and the regression to the mean phenomenon in order to be considered efficacious. Trials in client-owned animals can also be initiated earlier than human clinical trials, based on preclinical safety testing in dogs. In the present study, ES with use of a client questionnaire and use of rescue treatment were larger than objective measures of mobility such as activity monitoring and kinetic gait analysis. In addition, baseline CBPI was significantly correlated with other outcome measures at baseline. Clinical trials that use multiple objective and subjective outcome measures will yield the most robust test of drug efficacy.

## Methods

### Dogs

Forty nine client-owned medium to large breed dogs were prospectively enrolled in the study at the VMTH, University of Wisconsin-Madison. **Inclusion criteria** were history and clinical signs attributable to hip OA, including pelvic limb stiffness or lameness, abnormal hip range-of-motion, crepitation, and pain on joint manipulation, as well as radiographic evidence of OA in one or both hip joints. Hip radiographs were evaluated for periarticular osteophytes, enthesophytes, subchondral bone sclerosis, and remodeling of the femoral head and the acetabulum [41].

**Exclusion criteria** were history of surgery in the last 14 days, lameness of less than 4 weeks duration, and other pelvic limb conditions associated with joint instability, such as patella luxation and cranial (anterior) cruciate ligament rupture that had not been treated with a surgical stabilization procedure, neurological abnormalities, and concurrent major systemic disease.

### Ethics statement

Owners of dogs were required to sign informed consent forms before participation in the study. The study protocol was approved by the Institutional Animal Care & Use Committee, School of Veterinary Medicine, University of Wisconsin-Madison (V1448).

### Study design

A double-blind randomized placebo-controlled prospective clinical trial design was used that involved three visit days. At the start of the trial (Day 1), dogs were graded for clinical severity of lameness and pain (normal – 0; mild – 1; moderate −2; and severe - 3), and orthopaedic physical examination was performed. During recruitment of the last 12 dogs for the trial, group assignment was adjusted to ensure that clinical severity of lameness was equivalent in each treatment group. A key holder (PM), who did not participate in patient enrollment and allocation, generated the random sequence. Veterinarian investigators (SM, ZS, SS) undertook patient enrollment, allocation, and follow-up visits, after training by the principal investigator (PM). During physical examination, temperature, pulse rate, and respiratory rate (TPR) were recorded, thigh circumference was determined, and ROM was measured. Orthogonal radiographs of the pelvis were also obtained under sedation. If OA of another major joint was suspected clinically, additional radiographs were also made.

During the visit, the owner completed the CBPI questionnaire evaluating clinical status over the past week [29]. Force-plate analysis-of-gait was also performed. At the end of the day, the dog was discharged wearing an accelerometer located on the collar on the ventral part of the neck [32,42].

After a baseline week of no treatment, physical examination was repeated on Day 8, and each dog was randomly assigned to one of the 4 treatment groups (ABT-116, Carprofen, Tramadol, Placebo). The owners of dogs and the clinical investigator involved in client communication, patient assessment, and data collection were blinded to group assignment. A baseline peripheral venous blood sample was collected in a vacutainer tube (BD Vacutainer^TM^, Becton Dickinson, Franklin Lakes, NJ), force-plate analysis-of-gait was performed, and the first dose of medication was administered orally by the blinded investigator. Blood sampling was repeated 3 hours after treatment. Plasma was separated from the blood samples and stored at -20C. TPR and force-plate analysis-of-gait were also repeated 3 hours after dosing. The dog was then discharged at the end of day 8 and was given trial medication for 2 weeks by the owner in the home environment, after the owner completed a second CBPI baseline questionnaire.

On the third visit on day 22, 3 weeks after entering the trial, physical examination, blood sampling, and data collection were repeated as for Day 8, and the final dose of the trial medication was administered. The trial concluded at the end of Day 22 after the owner completed a third CBPI questionnaire regarding the dog’s clinical status during the second week of medication.

### Treatment and patient evaluation

A complete blood count and serum chemistry profile was performed if a dog was more than 7 years old, or if there were concerns with previous or concurrent medical conditions before trial enrollment. If dogs had been receiving treatment with an NSAID, a wash out period of 7 days was used before entry into the trial. This period was extended to 30 days if glucocorticoids had been administered [19]. If a dog had been given joint supplements, or was on medications that did not have any known analgesic or anti-inflammatory effects (e.g. phenylpropanolamine for urinary incontinence), administration of these compounds was continued during the trial. Treatment with nutritional supplements did not lead to exclusion from the trial and owners were instructed to continue habitual administration during the trial period.

### Analgesic medications

In this study, a TRPV1 inhibitor ABT-116 [1-[2-(3,3-dimethylbutyl)-4-(trifluoromethyl) benzyl]-3-(1-methyl-1 *H*-indazol-4-yl)urea] [43] (7.14% in Capmul PG-8 and Cremophor RH40 (9:1 w/w) solution) and two positive control drugs (NSAID and a synthetic opiate) were compared with placebo treatment (methylcellulose). TRPV1 is a ligand-gated non-selective cation channel expressed predominantly by nociceptive sensory neurons [44]. TRPV1 is primarily found in small-diameter primary afferent nerves, particularly unmyelinated C-fibers and Aδ fibers [45,46]. It integrates multiple pain stimuli such as noxious heat (> 42°), extracellular acidic pH < 5.7, and vanilloids such as capsaicin, which is a pungent ingredient of hot chili pepper [44,47,48]. Previous work has shown that TRPV1 antagonists have analgesic activity in various rodent models of chronic inflammatory and neuropathic pain [49-51]. Treatment with carprofen and tramadol in two additional groups was also used as positive controls. The NSAID carprofen is an effective treatment for canine OA that is widely used clinically [14,52]. Tramadol is an oral synthetic opiate medication that is also commonly used clinically for management of pain in dogs [53,54]. Dosage with ABT-116 was 3 mg/kg orally three times daily, dosage of carprofen was 2.2 mg/kg orally twice daily, dosage of tramadol was 4 mg/kg orally three times daily using immediate release tablets, and dosage of placebo was three times daily.

### Rescue treatment protocol

During the entire study period including both the baseline week and the two week treatment period, codeine/acetaminophen (1–2 mg/kg of codeine orally three times daily) was provided to the owners for use at home as a rescue analgesic. The owner recorded use of rescue therapy using a standard form. If rescue therapy was used within 48 hours of the second or third assessment day, force-plate analysis-of-gait was not performed [19].

### Outcome measures

#### Hip range-of-motion and thigh circumference measurements

Hip flexion and extension angles were measured using standard technique [55]. Measurement of mid-thigh circumference in each pelvic limb was also made using a tape measure device (Gulick II tape measure: Country Technology, Inc., Gays Mills, WI, USA).

#### Owner-completed canine brief pain inventory questionnaire

A validated client-specific outcome measures questionnaire was completed on each visit day [29]. In the questionnaire, owners were asked to provide an assessment of pain and the extent to which pain interfered with mobility exhibited by the dog in the previous week using a series of standardized questions. Clinical improvement in CBPI scoring was associated with a decrease in score over time.

#### Accelerometer measurement of activity

Dog activity was measured using a watch-sized, omnidirectional, accelerometer (Actical activity monitor, Mini Mitter Co Inc., Bend, OR) set to record in 1 minute epochs. The device was attached on Day 1 to the collar of each dog in a ventral position on the neck [32,42]. The device remained on the dog for the duration of the trial and was removed on Day 22. If owners removed the accelerometer for any reason, they were asked to record this. Data recorded during each visit day to the hospital (Days 1, 8, and 22) where excluded from analysis of activity. Total daily activity was divided into daytime activity (6 am to – 10 pm) and nighttime activity (10 pm – 6 am).

#### Force-plate analysis-of-gait

Force-plate analysis-of-gait was performed on Days 1, 8 and 22. The trials were done before sedation on the initial visit, and always before orthopaedic examination to avoid exacerbation of lameness. On Days 8 and 22, gait analysis was performed before administration of the trial medication, and was then repeated 3 hours after administration of the trial medication.

A biomechanical platform that measured 3-dimensional forces and impulses (OR6-6-1000 Biomechanics Platform with SGA6-4 Signal Conditioner/Amplifier, Advanced Mechanical Technologies Inc., Newton, MA) was used. The force plate was connected to a commercially available satellite data acquisition system to interface with the computer software used for gait analysis (Acquire v 7.30, from Sharon Software Inc., Dewitt, MI). On measurement days, dogs were trotted over the force plate using a leash. The force-plate system was calibrated for measurement of ground reaction forces using weights, and the photocells were calibrated for measurement of velocity using a pendulum. During each trial, the forward velocity of each dog was measured by 3 photoelectric cells mounted 1m apart on the gait analysis runway. The photocells were connected to a millisecond timer in a start–interrupt fashion. A handler guided each dog across the force plate, and an observer evaluated each pass across the plate to confirm foot strikes and gait. A minimum of 4 valid trials from each pelvic limb was considered acceptable for data analysis. A successful trial was defined by a forelimb hitting the plate followed by the ipsilateral pelvic limb within the predetermined velocity range of 1.3 – 1.9 m/s and an acceleration range of −0.5 m/s^2^ to +0.5 m/s^2^.

F_z_, VI, and FS were measured and recorded by the data acquisition system software. FS was defined as the slope of the straight line that connected the point of maximum force to the end of stance phase (zero force). It represents the rate at which a dog unloaded the limb. The FS is negative, and a larger negative value indicates that a dog was unloading the limb more rapidly (i.e. due to pain) [33]. All parameters were normalized by body weight. Data from the valid trials for each limb were averaged to obtain a mean value for each kinetic parameter. Only the results for the most severely affected limb were included in the analysis. The most severely affected pelvic limb selection was defined as the limb with the lowest F_z_.

### Measurement of drug plasma concentrations

Plasma was separated from blood samples by centrifugation and stored at -80C until analysis. ABT-116 and tramadol were evaluated using liquid/liquid extraction in tert-butyl methyl ether (TBME), and injected in a Thermo BetaBasic CN 50 × 3 mm column on an AbSciex N API-2000 TIS TurboSpray LC-MS mass spectrometry system (Foster City, California) for analysis. Carprofen was extracted with acetonitrile, and injected in a Thermo BetaBasic CN 50 × 3 mm column on an AbSciex N API-4000 TIS TurboSpray LC-MS (Foster City, California) for analysis.

### Statistical analysis

The Kolmogorov-Smirnov test was used to estimate whether data approximated a normal distribution. Data were reported as mean ± standard deviation or median (range) as appropriate. The Kruskal-Wallis ANOVA was used to determine whether clinical severity of lameness at trial entry was influenced by group. Body weight and age were compared between groups using one-way ANOVA. Repeated-measures ANOVA was used to analyze the effect of trial medication on rectal temperature, hip ROM, and thigh circumference. Mean baseline ROM and thigh circumference (average of the values obtained on Day 1 and Day 8) were compared with values obtained at the end of the trial. Changes from mean baseline (average of the values obtained on Day 1 and Day 8) in total CBPI, pain severity, and pain interference scores after treatment were analyzed using one-way ANOVA and the single-sample Student’s *t* test against a hypothesized mean of zero (no change). Change in dog activity between Weeks 1 and 3 was examined using one-way ANOVA, the single-sample Student’s *t* test, or the Wilcoxon test. Change from mean baseline in F_z_, VI, and FS after treatment was analyzed using the single-sample Student’s *t* test. Use of rescue medication during each week of the trial was assessed using the Kruskall-Wallis ANOVA, Friedman ANOVA, and Wilcoxon tests. The Student’s *t* test for paired data was used to compare plasma drug concentrations on Day 8 and Day 22. Relationships between age, weight, gender, and outcome measures of mobility at baseline and after treatment were examined using the Spearman Rank correlation statistic. Results were considered significant at *P* < 0.05. A Dunn-Sidak correction was used for multiple comparisons as necessary (significance at *P* < 0.01274).

Placebo effects were defined as the overall change from baseline in the placebo group. Placebo effect was estimated as the effect size (ES: the standard mean difference between baseline and end-point). ES for parametric data was calculated using the Cohen’s d method [56]. ES for non-parametric data was estimated using the Cliff’s Delta method [57]. Clinically, an ES of ≤ 0.2 suggests a small effect, > 0.2 to < 0.8 a moderate effect, and ≥ 0.8 a large effect [56]. Correspondence analysis was also used to explore the influence of analgesic treatment group on the pattern of CBPI questionnaire responses.

## Abbreviations

OA: Osteoarthritis; TRPV1: Transient receptor potential vanilloid 1; NSAID: Non-steroidal anti-inflammatory drug; DMOAD: Disease modifying osteoarthritic drug; ES: Effect size; VMTH: University of Wisconsin-Madison Veterinary Medical Teaching Hospital; ROM: Range-of-motion; CBPI: Canine brief pain inventory; Fz: Peak vertical force; VI: Vertical impulse; FS: Falling slope; ABT-116: 1-[2-(3,3-dimethylbutyl)-4-(trifluoromethyl) benzyl]-3-(1-methyl-1*H*-indazol-4-yl)urea; TPR: Temperature, pulse, respiration; TBME: Tert-butyl methyl ether.

## Competing interests

Peer Jacobson and Elizabeth Cozzi are stockholders and employees of Abbott Laboratories. Abbott Laboratories assisted with measurement of plasma drug concentrations and provided funding to the Comparative Orthopaedic Research Laboratory for basic research on development of a clinical trial model for assessment of analgesic therapy for arthritis pain in client-owned dogs that included assessment of efficacy of ABT-116. ABT-116 is a patented compound (WO 2008/024945 A1 02-28-2008) that was developed by Abbott Laboratories and was used as one of three test articles in this study. The author(s) declare that they have no competing interests.

## Authors' contributions

PM, PBJ and EMC conceived and designed the experiments. SM, SJS, ZS, BN, PBJ, EMC, JAB, SLS, GH and PM performed the experiments. PBJ and EMC contributed reagents and analysis tools. SM and PM analyzed the data. SM and PM wrote the paper. All authors read and approved the final manuscript.
